# A Rare Case of Risperidone-Induced Hypothermia and Thrombocytopenia

**DOI:** 10.7759/cureus.24836

**Published:** 2022-05-09

**Authors:** Lokesh Goyal, Raymond Acebo, Shah Islam, Kunal Ajmera, Sriveer Kaasam

**Affiliations:** 1 Hospital Medicine, CHRISTUS Spohn Hospital Corpus Christi - Shoreline, Corpus Christi, USA; 2 Internal Medicine, CHRISTUS Spohn Hospital Corpus Christi - Shoreline, Corpus Christi, USA; 3 Nephrology/Internal Medicine, CHRISTUS Spohn Hospital Corpus Christi - Shoreline, Corpus Christi, USA; 4 Epidemiology, George Washington University, Washington, D.C., USA

**Keywords:** hypothermia, atypical antipsychotic, risperidone, schizophrenia and other psychotic disorders, clinical psychiatry

## Abstract

Psychosis is a term defined in medical literature broadly as someone who has lost contact with reality. Psychosis is typically seen in multiple psychiatric disorders, for example, schizophrenia, bipolar disorder, and severe depression. It is also seen in patients abusing drugs and other underlying medical conditions like hepatic impairment, renal failure, etc. Typically, patients with psychosis will present with hallucinations, delusions, disorganized speech, and behavior. Patients with psychosis are usually treated with antipsychotic medications. There are two types of antipsychotics: typical and atypical antipsychotics. Typical antipsychotics have a low safety profile and are associated with side effects like pancytopenia, hyperthermia, and hypothermia. Therefore, physicians and other medical professionals try to avoid prescribing typical antipsychotics for long-term use. Risperidone, an atypical antipsychotic, is considered relatively safe in patients compared to other antipsychotics. This case study will see risperidone causing hypothermia and thrombocytopenia in a healthy 34-year-old patient.

## Introduction

Typical and atypical antipsychotic medications are very effective in the treatment of acute and chronic psychosis. Atypical antipsychotics, also known as second-generation antipsychotics, have lower extrapyramidal side effects when compared to typical antipsychotics. Although both the first and second generations of antipsychotics are comparable in their efficacy, atypical antipsychotics like risperidone tend to have better efficacy in the treatment of schizophrenia because it has fewer extrapyramidal side effects and a safe hematological profile [1].

Second-generation (atypical) antipsychotics tend to have a higher affinity towards serotonin binding receptor, 5-HT2, than dopamine receptors, D2. In first-generation (typical) antipsychotics, we typically see a higher affinity toward dopamine receptors D2. Therefore, because of the affinity of atypical antipsychotics towards serotonin receptors, they have lower extrapyramidal side effects when compared to first-generation antipsychotics [1,2].

Risperidone is primarily used to treat schizophrenia, but it can also be used to treat aggression, bipolar disorder, agitation, and aggression. In this case report, we will focus on risperidone causing hypothermia and thrombocytopenia, which is very uncommon but is a dangerous side effect of this medication [3,2].

## Case presentation

The patient, a 34-year-old male with a past medical history of bipolar disorder, schizophrenia, and seizure disorder on Keppra, presented to the emergency room from prison (where he has been detained in the mental health ward since August 2021) with concerns of altered mental status for the past 24 hours. The patient is alert and tracking but not following commands or answering questions. He was found to have hypothermia of 88 °F and severe thrombocytopenia. The vital signs are given in Table 1 and laboratory values in Table 2.

**Table 1:** Vital signs of the patient

	Normal Range	April 10, 2022	April 11, 2022	April 12, 2022	April 13, 2022	April 14, 2022
Temperature (in Fahrenheit or °F)	97 °F - 98.9 °F	88.0 °F (Low)	94.2 °F (Low)	97.2 °F	98.1 °F	98.0 °F
Pulse	60-100	42 (Low)	58 (Low)	66	74	82
Blood Pressure (mmHg)	Systolic: 110-139, Diastolic 60-89	86/40 (Low)	98/60 (Low)	114/65	118/74	124/82
O2 Delivery Method	Room air	Room air	Room air	Room air	Room air	Room ai

**Table 2:** Laboratory test values of the patient

	Normal Range	April 10, 2022	April 11, 2022	April 12, 2022	April 13, 2022	April 14, 2022
White Blood Cells	4.5 to 11.0 × 10*9/L	6.98	7.52	8.62	7.88	7.92
Hemoglobin	13.2 to 16.6 gm/dL	13.5	12.5 (low)	13.2 (low)	12.9 (low)	12.8 (low)
Platelet Count	150 to 400 × 10*9/L	39 (Low)	28 (Low)	38 (low)	80 (low)	141 (normal)
Thyroid Stimulating Hormone	0.5 to 5.0 mIU/L	4.2				
Comprehensive Metabolic Panel	Normal	Normal				
Cortisol Level (AM)	Normal	Normal				
Urinalysis	Normal	Normal				
Urine Drug Screen	Normal	Normal				
Blood Culture X2	Normal	Normal				
Lumbar Puncture	Normal	Normal				

The medical records were obtained from the prison, showing that the patient was started on risperidone therapy almost six weeks ago. The patient has lived in the prison's medical ward for four months because of his schizophrenia. His symptoms began recently, almost six weeks after starting his risperidone therapy. He became hypothermic, thrombocytopenic, and slightly altered. The patient had a urine drug screen, which was negative. CT head showed no acute abnormality. He was admitted to the ICU for further evaluation and treatment.

In the ICU, the patient was noted to be slightly hypotensive; therefore, Levophed was started to help with the patient's blood pressure. Levophed was eventually weaned off and discontinued. Neurology was consulted on this patient. MRI was performed, which was completely normal. The patient had negative blood cultures, urine cultures, and a normal chest x-ray. An echocardiogram was also performed, showing normal ejection fraction with grade 1 diastolic dysfunction. A lumbar puncture was also performed, which was normal as well.

The patient's home medication included Keppra 500 twice daily, risperidone 3 mg three times daily, and trazodone 100 mg at bedtime as needed for sleep. Psychiatry was consulted for the patient's schizophrenia and they recommended discontinuation of the patient's risperidone. The patient's hypothermia resolved after 48 - 72 hours of discontinuation of his medication. The patient's thrombocytopenia and hypothermia resolved completely three days after discontinuation of the patient's risperidone. His mental status returned to his baseline as well. The patient was then transferred out of ICU to the medical ward, where he was observed for one more day and then discharged.

## Discussion

Atypical antipsychotics like risperidone are classically used to treat schizophrenia, bipolar disorder, aggressive behavior, etc. Risperidone is preferred because of its better safety profile than other atypical antipsychotics. It acts as an antagonist to both 5-HT2 (serotonin) and D2 (dopamine) receptors but more so on the 5-HT2, which allows it to have a lower incidence of extrapyramidal side effects [1].

The exact mechanism of hypothermia associated with risperidone or any other antipsychotic medications is not very well known. However, there have been several hypotheses, out of which the most important one described by Van Marum and colleagues [2] states that risperidone has a higher affinity to serotonin receptors compared to dopamine, and since serotonin is associated with thermoregulation, therefore, risperidone is associated with hypothermia. Also, risperidone blocks the alpha-2 adrenergic receptor, which is involved in thermoregulation. Alpha-2 adrenergic receptor causes vasoconstriction and shivering during cold and, therefore, by blocking the alpha-2 adrenergic receptors, risperidone can cause hypothermia [3]. Thrombocytopenia is also a rare side effect of an antipsychotic medication like risperidone, leading to life-threatening complications. Studies show that it affects platelet aggregation [4]; however, the exact mechanism remains unclear [5].

Treatment of antipsychotic-induced hypothermia included noninvasive external rewarming like blankets, warm forced air, and the use of hot water bottles. Once the offending agent, risperidone, was removed, the patient's hypothermia wholly resolved in about 48-72 hours. The treatment of antipsychotic-induced thrombocytopenia (when the patient is not actively bleeding) is to remove the offending antipsychotic agent. If the patient is actively bleeding, platelet infusion through IV is necessary. This technique was influential in the treatment of this patient [6].

## Conclusions

Hypothermia and thrombocytopenia are rare and severe side effects of risperidone, which can be life-threatening if not identified and treated appropriately. Risperidone is used extensively in both inpatient and outpatient settings worldwide. Early recognition and identification of these side effects will lead to earlier treatment and recovery of the patient. 
